# Observations on the Connexion between Famine and Fever in Ireland and Elsewhere

**Published:** 1847-07

**Authors:** 


Art. VII.—*Observations on the Connexion between Famine and Fever in Ireland and elsewhere.* By Henry Kennedy, a.b., m.b., &c.—1847. Pamphlet, 8vo, pp. 50.

These observations are a reply to Dr. Corrigan's pamphlet, on Famine and Fever as Cause and Effect in Ireland. Dr. Corrigan's facts are analysed and shown to have a different meaning than that which he has drawn from them, while his doctrine, that famine is the prevalent cause of fever in Ireland, is shown to be untenable. It is evident that Dr. Kennedy has the best of the argument, partly because he takes a wider view of the causes of epidemical diseases, partly because he has apparently a more philosophical turn of mind than his antagonist, and partly because Dr. Corrigan's position was one of the most untenable he could take up.

Dr. Kennedy maintains that the use of bad or deficient food injures the health by weakening the vital powers, and if the quantity be too small to maintain these diminished vital powers, then death results. This unhealthy condition predisposes to the attacks of diseases, and of fever amongst the rest, and if an epidemic be prevalent will increase its fatality, and prevalence, like any other debilitating cause. The destruction of vegetable life causing the famine, is dependent on the same cause as the epidemic itself; that, in fact, there is an "epidemic constitution" which involves all organisms within its influence, so that not only are these epidemical diseases amongst men, but domestic and other animals suffer with them. All this is, we think, very reasonable and in accordance with the views of the best writers on the subject.

It is manifest that a proper understanding of the meaning of terms used, is of first importance in a discussion of this kind. We are sure Dr. Corrigan would grant the utmost importance in the etiology of the Irish epidemic to the habits of the Irish people. In what other country in the civilized world do we read of corpses being left unburied in the filthy hovels in which the inhabitants have died? of the dead being devoured by dogs, simply because their graves are indolently made so shallow as to be within a few inches only of the surface? of the awful crowding in the hovels and workhouses? of the piling up of coffins outside a union workhouse, containing two, three, or four corpses each; remaining there for a fortnight unburied, and sweltering in putrefaction and decay? There is not a single practitioner in England and Scotland, who, with such facts before him, would say that a want of food was the cause of fever in a country like Ireland, and among a people like the Irish. Dr. Corrigan will be justified in stating that famine causes the over-crowding of the hovels, hospitals, and workhouses, but surely there are more direct causes of fever *continually* existing in Ireland than an empty stomach.

The inundation of the starving population of Ireland has convinced the inhabitants of Great Britain that the masses have no regard to personal cleanliness, or the decencies of civilized life. So long as the Irish continue in that condition, every successive epidemic must necessarily be destructive, and the well-meaning philanthropic efforts of men like Dr. Corrigan remain unregarded.

